# Network pharmacology and molecular docking analysis on the mechanism of Wensan tincture in the treatment of pulmonary nodules: A review

**DOI:** 10.1097/MD.0000000000040648

**Published:** 2024-11-29

**Authors:** Jinzhi Zhang, Jingqi Yang, Guangxi Li

**Affiliations:** a Respiratory Department, Guang’anmen Hospital, China Academy of Chinese Medical Sciences, Beijing, China; b Respiratory Department, Xiyuan Hospital, China Academy of Chinese Medical Sciences, Beijing, China.

**Keywords:** molecular docking, network pharmacology, pulmonary nodules, signaling pathway, Wensan tincture

## Abstract

Network pharmacology and molecular docking methods were applied to elucidate the molecular mechanism of action of Wensan tincture (WST) in the treatment of pulmonary nodules. The Traditional Chinese Medicine Systems Pharmacology and the Traditional Chinese Medicine and Chemical Composition database were used to screen the active ingredients. Potential targets of WST were retrieved using Traditional Chinese Medicine Systems Pharmacology, SwissADME, and SwissTargetPrediction, while pulmonary nodule-associated targets were obtained from GeneCards and Online Mendelian Inheritance in Man databases. An active ingredient–target network was constructed using Cytoscape 3.9.1, and Gene Ontology and Kyoto Encyclopedia of Genes and Genomes enrichment analyses were conducted via the Database for Annotation, Visualization, and Integrated Discovery platform to identify core targets and signaling pathways. Molecular docking studies were performed using AutoDockTools. The results revealed 62 active ingredients and 344 corresponding targets within the tincture, alongside 1005 targets associated with pulmonary nodules. Gene Ontology and Kyoto Encyclopedia of Genes and Genomes enrichment analyses indicated that the potential therapeutic targets of WST include signal transducer and activator of transcription 3, mitogen-activated protein kinase-3, mitogen-activated protein kinase-1, Jun proto-oncogene, tumor protein 53, phosphoinositide-3-kinase regulatory subunit 1, heat shock protein 90 alpha family class A member 1, and AKT serine/threonine kinase 1. The primary pathways were the cancer pathway, mitogen-activated protein kinase signaling, advanced glycation end-products and their receptor signaling, epidermal growth factor receptor signaling, hypoxia-inducible factor-1 signaling, and the programmed cell death-ligand 1/programmed cell death protein 1 checkpoint pathways. Molecular docking demonstrated that quercetin exhibited the strongest binding affinity with mitogen-activated protein kinase-3, with a binding energy of −9.1 kcal/mol. Notably, key components of WST, such as quercetin, demonstrate considerable potential as drug candidates for the treatment of pulmonary nodules.

## 1. Introduction

A pulmonary nodule is an abnormal area in the lung measuring <3 cm in diameter.^[1]^ Pulmonary nodules may be solitary or multiple but not accompanied by atelectasis, hilar lymph node enlargement and pleural effusion.^[2]^ With the widespread use of low-dose computed tomography and the increased screening of respiratory disease patients during the COVID-19 pandemic, the detection rate of pulmonary nodules has risen.^[2]^ Data from the United States indicate that the incidence of incidentally detected pulmonary nodules is 5.8 per 100,000 person-years in women and 5.2 per 100,000 person-years in men.^[3]^ Cross-sectional studies revealed that the detection rate of pulmonary nodules in screening populations ranges from 2.1% to 64.5%, with early-stage lung cancer detection rates ranging from 0.45% to 2.1%.^[4–6]^ The pathogenesis of pulmonary nodules is associated with age, genetics, smoking and secondhand smoke exposure, dust exposure, and previous chronic lung diseases,^[7]^ but the underlying mechanisms are still not clear. Patients with pulmonary nodules typically exhibit no specific pulmonary symptoms and often present with comorbid conditions as the primary manifestations. Western medicine treatment mainly includes 2 categories, one is surgical resection, the other is nonsurgical therapy, such as radiofrequency ablation. Since most incidental or screen-detected pulmonary nodules are benign lesions or indolent lung cancer nodules, the comparison of early surgical intervention with follow-up elective surgery did not yield a significant improvement in overall patient survival.^[8]^ Consequently, regular surveillance and follow-up observation have emerged as the primary recommended management approach for the vast majority of pulmonary nodules. For nodules with a high likelihood of being benign, the preferred approach involves regular follow-up with chest CT. However, the uncertainty regarding the progression of pulmonary nodules imposes a considerable psychological burden on patients.^[9]^ Nearly half of respondents experience emotional distress 6 to 8 weeks after the identification of pulmonary nodules.^[10]^ Therefore, strategies are needed to alleviate this distress, particularly for younger patients, women, current smokers, individuals from minoritized groups, and those with larger nodules.^[10]^ Chinese medicine originally categorizes lung nodules as “phlegm nuclei” and “lung stagnation.”^[11]^ Wensan tincture (WST) is a traditional Chinese medicine compound preparation developed by Guang’anmen Hospital, known for its effects of “warming the meridians, promoting blood circulation, removing stasis, and resolving phlegm.” While it is widely used in the clinical treatment of pulmonary nodules, the underlying mechanisms of this formulation remain unclear.

**Figure 1.**
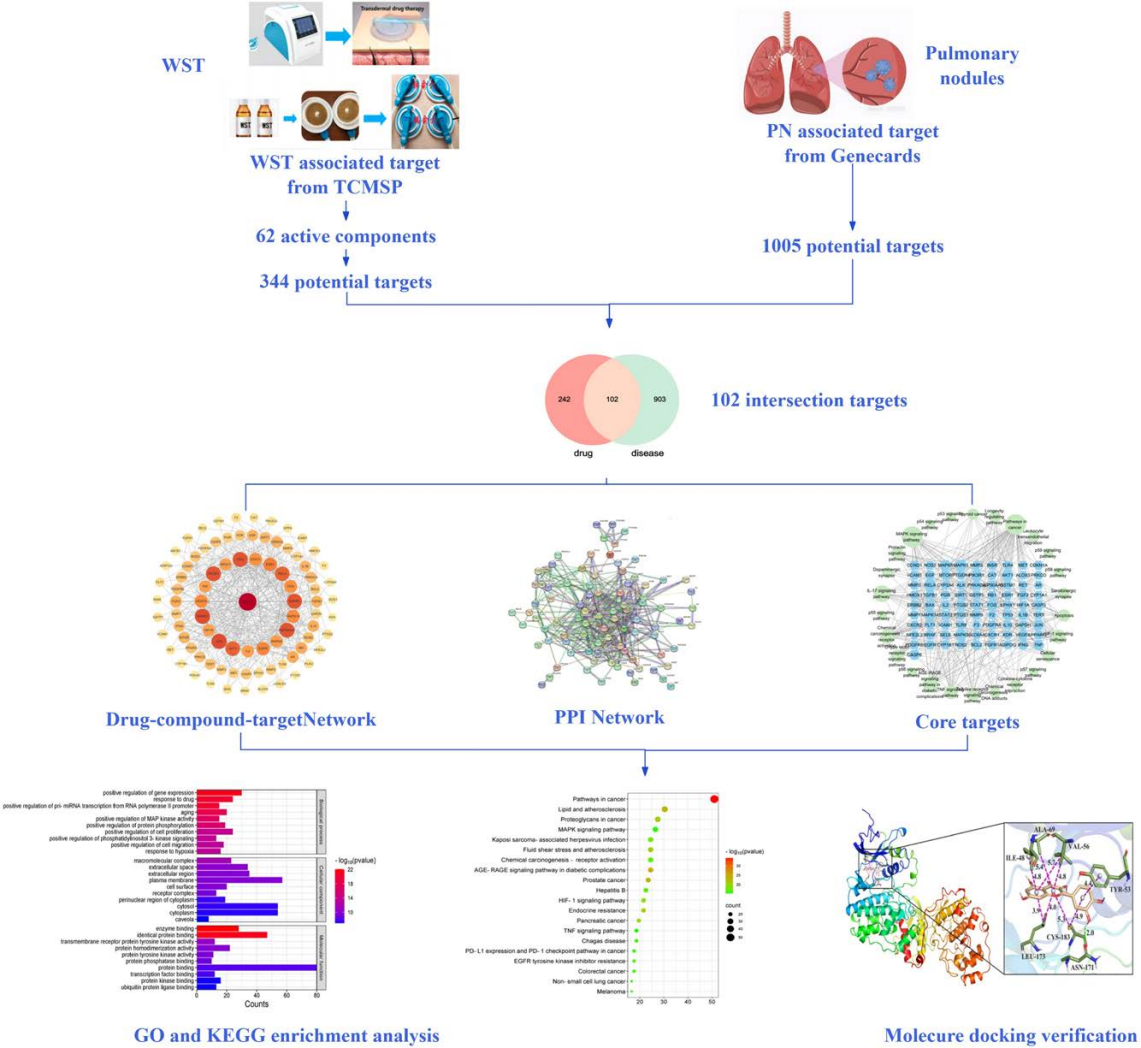
The study design and workflow diagram.

Network pharmacology enables the analysis of the relationships between the active ingredients of drugs and diseases by examining biological networks, thus clarifying their mechanisms of action.^[12]^ In this study, we will use the network pharmacology research method to explore the potential targets and mechanism of action in the treatment of pulmonary nodules, and use molecular docking technology to verify it, in order to provide theoretical basis and reliable data support for subsequent studies. We applied network pharmacology to systematically analyze the potential mechanism, predict the core targets and pathways of WST in treating pulmonary nodules. The study design and workflow diagram are shown in Figure 1.

## 2. Materials and methods

### 2.1. Screening of active ingredients and target genes in WST

Using the Traditional Chinese Medicine Systems Pharmacology (https://old.tcmsp-e.com/tcmsp.php) database with oral bioavailability ≥ 30% and drug-like properties ≥ 0.18 as the screening conditions, we obtained the active ingredients and targets of Tiannanxing, Weilingxian, Duhuo, Tufuling, Chuanxiong, Danggui, Xixin, Zhimaqianzi, and Zhichuanwu. The traditional Chinese medicine and chemical component databases were utilized to identify the active ingredients of Shengdihuang and obtain their Chemical Abstracts Service numbers. The active ingredients of WST were entered into the PubChem database (https://pubchem.ncbi.nlm.nih.gov/) to retrieve their chemical structures. The Swiss ADME database (http://www.swissadme.ch) was used to screen drug components. By inputting the drug ingredients in Structure Data Files, we selected the relevant drug ingredient to fulfill Absorption high and Druglikeness (Lipinski, Ghose, Veber, Egan, Muegge, etc) for 2 out of any 5 Yes items. The Swiss Target Prediction (http://www.swisstargetprediction.ch/) database was used to obtain the relevant targets. Finally all the drug targets obtained were validated using the UniProt database (http://www.uniprot.org/).

### 2.2. Construction of drug–effective active ingredient–target network

Each Chinese herb medicine was input into the Excel table in 2nd columns in the form corresponding to its active ingredients and targets, and then the information was imported into Cytoscape 3.9.1 software to construct the network map of the Chinese medicine compound–effective active ingredient–target.

### 2.3. Collection of targets related to pulmonary nodules

The Online Mendelian Inheritance in Man database (https://www.omim.org/) and the Gene Comprehensive Database, GeneCards (https://www.genecards.org/) were used to identify relevant genes associated with pulmonary nodules by utilizing specific keywords, including “pulmonary nodule,” “pulmonary-nodule,” and “pulmonary nodules.” Pulmonary nodule-related targets were collected, and duplicate entries were removed.

### 2.4. Screening of the potential targets of WST for the treatment of pulmonary nodules

With the Venny 2.1.0 software (https://bioinfogp.cnb.csic.es/tools/venny/index.html), a Venn diagram was constructed to obtain potential shared target points for treating pulmonary nodules with WST.

### 2.5. Protein–protein interaction network construction and topology analysis

The intersecting targets were subsequently imported into the STRING database (https://string-db.org) to construct a protein–protein interaction (PPI) network. Key targets were identified using Cytoscape 3.9.1 software by selecting nodes with degree values, betweenness centrality, and closeness centrality metrics that were equal to or greater than the average.

### 2.6. GO functional annotation of intersecting targets and KEGG pathway enrichment analysis

Gene Ontology (GO) and Kyoto Encyclopedia of Genes and Genomes (KEGG) enrichment analyses were performed using the Database for Annotation, Visualization, and Integrated Discovery to elucidate the biological functions and pathways associated with the identified targets. The results were visualized using advanced bioinformatics software, with findings presented as bar graphs and bubble plots, respectively.

### 2.7. Component–core target molecular docking validation

The PDB database (https://www.rcsb.org/) and relevant literature, along with the UniProt database, were utilized to obtain the PDB structure of the docking protein. Preparatory operations, including desolvation and hydrogenation, were conducted using AutoDockTools-1.5.6 (https://autodock.scripps.edu/resources/), with the resulting data saved in PDBQT format. Molecular structure files for the core active ingredients were retrieved from the Traditional Chinese Medicine Systems Pharmacology database, and AutoDockTools-1.5.6 was employed to balance charges and identify rotatable bonds in the small molecules, which were also saved in PDBQT format. These PDBQT files were imported into AutoDockTools-1.5.6, where the docking box was defined according to the receptor’s active site. The binding energy, quantified as a negative value, indicated that larger magnitudes correspond to greater binding affinity and enhanced complex stability, suggesting a higher likelihood of interaction between receptor molecules and ligands. The structure with the lowest binding energy was exported as a PDB file and imported into PyMOL 2.5.4 for optimization, ultimately generating the final visualization. Interactions between receptor proteins and small molecules were illustrated in a two-dimensional structural diagram.

## 3. Results

### 3.1. Active ingredients of WST and their action targets

A total of 74 active ingredients were screened from WST, including 14 from Zhimaqianzi, 3 from Zhichuanwu, 7 from Tiannanxing, 7 from Weilingxian, 9 from Duhuo, 15 from Tufuling, 7 from Chuanxiong, 2 from Danggui, 8 from Xixin, and 13 from Shengdihuang. Among these, 62 active ingredients were successfully matched to target sites in the database. Of these, Shengdihuang accounts for 11, while the remaining 51 active components are detailed in Table 1. The corresponding target proteins were then input into the UniProt database. After eliminating invalid and duplicate targets, a total of 344 unique targets associated with the active components of WST were identified.

**Table 1:** Bioactive compounds of Wensan tincture.

MOL ID	Effective active ingredients	OB ≥ 30%	DL ≥ 0.18	Drug source	ID
MOL001040	(2*R*)-5,7-dihydroxy-2-(4-hydroxyphenyl)chroman-4-one	42.36	0.21	Zhimaqianzi	MQZ1
MOL001476	(*S*)-Stylopine	51.15	0.85	Zhimaqianzi	MQZ2
MOL003411	Icaride A	48.74	0.43	Zhimaqianzi	MQZ3
MOL003413	Isostrychnine N-oxide (I)	35.45	0.8	Zhimaqianzi	MQZ4
MOL003414	Isostrychnine N-oxide (II)	37.33	0.8	Zhimaqianzi	MQZ5
MOL003418	Lokundjoside_qt	32.82	0.76	Zhimaqianzi	MQZ6
MOL003432	Vomicine	47.56	0.65	Zhimaqianzi	MQZ7
MOL003433	Brucine-N-oxide	49.17	0.38	Zhimaqianzi	MQZ8
MOL003436	Isobrucine	33.58	0.8	Zhimaqianzi	MQZ9
MOL003440	Brucine-N-oxide	52.63	0.38	Zhimaqianzi	MQZ10
MOL000449	Stigmasterol	43.83	0.76	Zhimaqianzi, Tufuling, Tiannanxing, Weilingxian, Danggui	A1
MOL000492	(+)-Catechin	54.83	0.24	Zhimaqianzi	MQZ12
MOL013117	4,7-Dihydroxy-5-methoxyl-6-methyl-8-formyl-flavan	37.03	0.28	Tufuling	TFL1
MOL013118	Neoastilbin	40.54	0.74	Tufuling	TFL2
MOL013119	Enhydrin	40.56	0.74	Tufuling	TFL3
MOL013129	(2*R*,3*R*)-2-(3,5-dihydroxyphenyl)-3,5,7-trihydroxychroman-4-one	63.17	0.27	Tufuling	TFL4
MOL001736	(‐)-Taxifolin	60.51	0.27	Tufuling, Danggui, Tiannanxing, Duhuo, Weilingxian	B1
MOL000358	beta-Sitosterol	36.91	0.75	Tufuling, Tiannanxing, Chuanxiong	C1
MOL000359	Sitosterol	36.91	0.75	Tufuling	TFL7
MOL000449	Stigmasterol	43.83	0.76	Tufuling	TFL9
MOL004567	Isoengelitin	34.65	0.7	Tufuling	TFL10
MOL004575	Astilbin	36.46	0.74	Tufuling	TFL11
MOL004576	Taxifolin	57.84	0.27	Tufuling	TFL12
MOL004580	cis-Dihydroquercetin	66.44	0.27	Tufuling	TFL13
MOL000546	Diosgenin	80.88	0.81	Tufuling	TFL14
MOL000098	Quercetin	46.43	0.28	Tufuling	TFL15
MOL002086	1-[(5*R*,8*R*,9*S*,10*S*,12*R*,13*S*,14*S*,17*S*)-12-hydroxy-10,13-dimethyl-2,3,4,5,6,7,8,9,11,12,14,15,16,17-tetradecahydro-1H-cyclopenta[a]phenanthren-17-yl]ethanone	33.47	0.42	Zhichuanwu	CW1
MOL002087	delta-4,16-Androstadien-3-one	37.63	0.31	Zhichuanwu	CW2
MOL013156	[(2*R*)-2-[[[(2*R*)-2-(benzoylamino)-3-phenylpropanoyl]amino]methyl]-3-phenylpropyl] acetate	38.88	0.56	Tiannanxing	TNX1
MOL001510	24-Epicampesterol	37.58	0.71	Tiannanxing	TNX2
MOL000953	Cholesterol	37.87	0.68	Tiannanxing	TNX6
MOL005603	Heptyl phthalate	42.26	0.31	Weilingxian	WLX3
MOL001941	Ammidin	34.55	0.22	Duhuo	DH1
MOL001942	Isoimperatorin	45.46	0.23	Duhuo	DH2
MOL004777	Angelol D	34.85	0.34	Duhuo	DH4
MOL004778	[(1*R*,2*R*)-2,3-dihydroxy-1-(7-methoxy-2-oxochromen-6-yl)-3-methylbutyl] (*Z*)-2-methylbut-2-enoate	46.03	0.34	Duhuo	DH5
MOL004780	Angelicone	30.99	0.19	Duhuo	DH6
MOL004792	Nodakenin	57.12	0.69	Duhuo	DH7
MOL002135	Myricanone	40.6	0.51	Chuanxiong	CX2
MOL001494	Mandenol	42	0.19	Chuanxiong	CX3
MOL002157	Wallichilide	42.31	0.71	Chuanxiong	CX4
MOL002140	Perlolyrine	65.95	0.27	Chuanxiong	CX5
MOL000433	Ferulic acid	68.96	0.71	Chuanxiong	CX6
MOL009849	ZINC05223929	31.57	0.83	Xixin	XX1
MOL012141	Caribine	37.06	0.83	Xixin	XX2
MOL000422	Kaempferol	41.88	0.24	Xixin	XX3
MOL002962	(3*S*)-7-hydroxy-3-(2,3,4-trimethoxyphenyl)chroman-4-one	48.23	0.33	Xixin	XX4
MOL001558	Sesamin	56.55	0.83	Xixin	XX5
MOL002501	[(1*S*)-3-[(*E*)-but-2-enyl]-2-methyl-4-oxo-1-cyclopent-2-enyl] (1*R*,3*R*)-3-[(*E*)-3-methoxy-2-methyl-3-oxoprop-1-enyl]-2,2-dimethylcyclopropane-1-carboxylate	62.52	0.31	Xixin	XX6
MOL012140	4,9-Dimethoxy-1-vinyl-$b-carboline	65.3	0.19	Xixin	XX7
MOL001460	Cryptopin	78.74	0.72	Xixin	XX8

OB = oral bioavailability, DL = drug-like properties.

### 3.2. Construction of drug–effective active ingredient–target network

**Figure 2.**
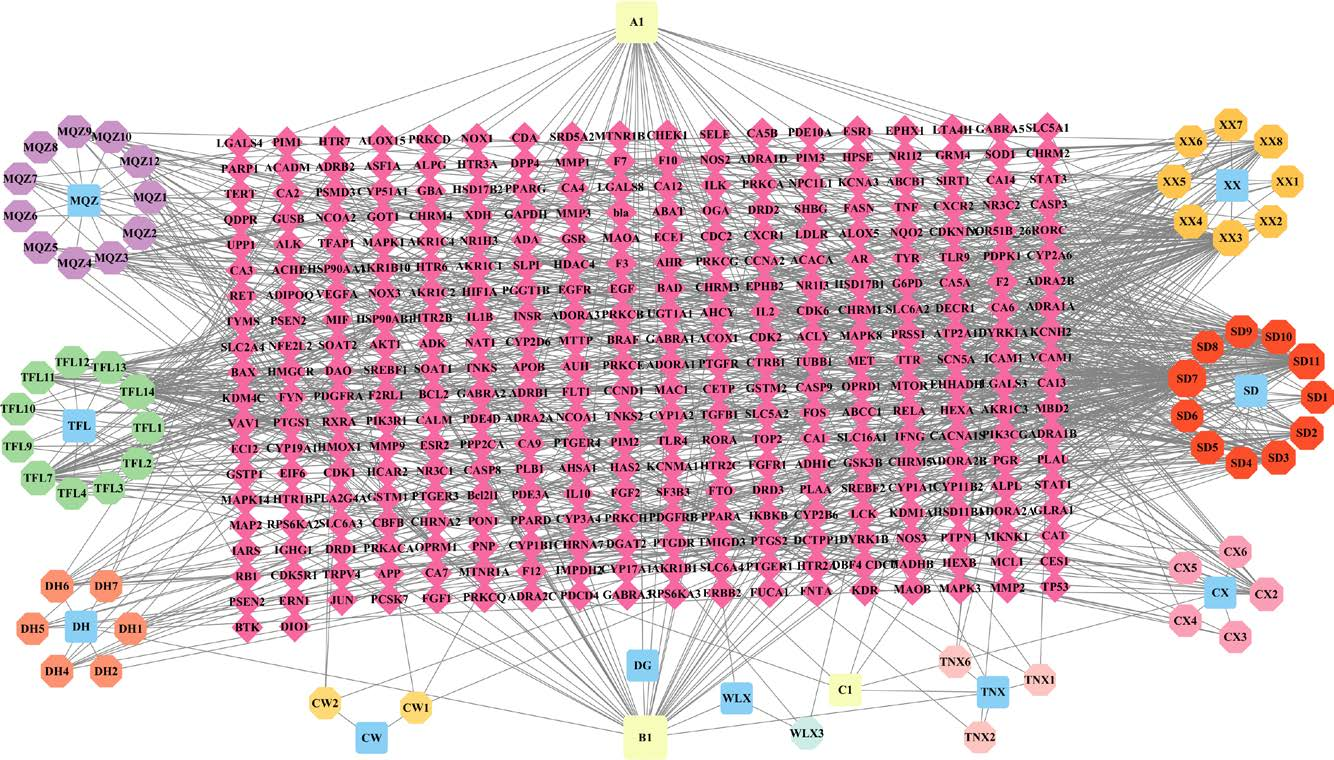
Drug–effective active ingredient–target network diagram.

The active ingredients and their corresponding targets were imported into Cytoscape 3.9.1 software to design network diagrams that illustrate potential interactions between various active ingredients and core targets, forming the drug–active ingredient–target network. A total of 415 nodes and 904 interaction relationships were identified, as depicted in Figure 2. The top 5 compounds based on their degree values were β-sitosterol, stigmasterol, kaempferol, quercetin, and spiroside, as detailed in Table 2.

**Table 2:** The top 5 compounds by degree.

ID	MOL ID	Chemical composition name	Degree price	OB	DL
B1	MOL000358	beta-Sitosterol	195	36.91	0.75
A1	MOL000449	Stigmasterol	160	43.83	0.76
SD7	MOL003341	Salidroside	88	40.12	0.2
XX3	MOL000422	Kaempferol	62	41.88	0.24
TFL15	MOL000098	Quercetin	46	46.43	0.28

OB = oral bioavailability, DL = drug-like properties.

### 3.3. Key targets of action of WST for the treatment of pulmonary nodules

**Figure 3.**
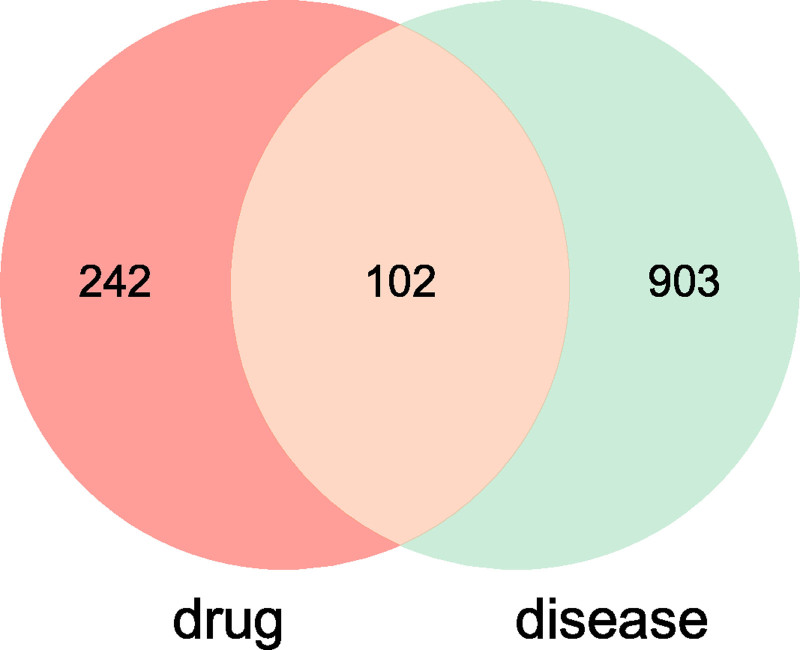
Drug–disease target Venn diagram.

Through the integration of data from the Online Mendelian Inheritance in Man, GeneCards, and DisGeNet databases, a total of 1004 targets associated with pulmonary nodules were retrieved. The intersection of these targets with the 344 active component targets of WST resulted in 178 key targets (common targets) for the treatment of pulmonary nodules. The results are presented in Figure 3.

### 3.4. PPI network construction

**Figure 4.**
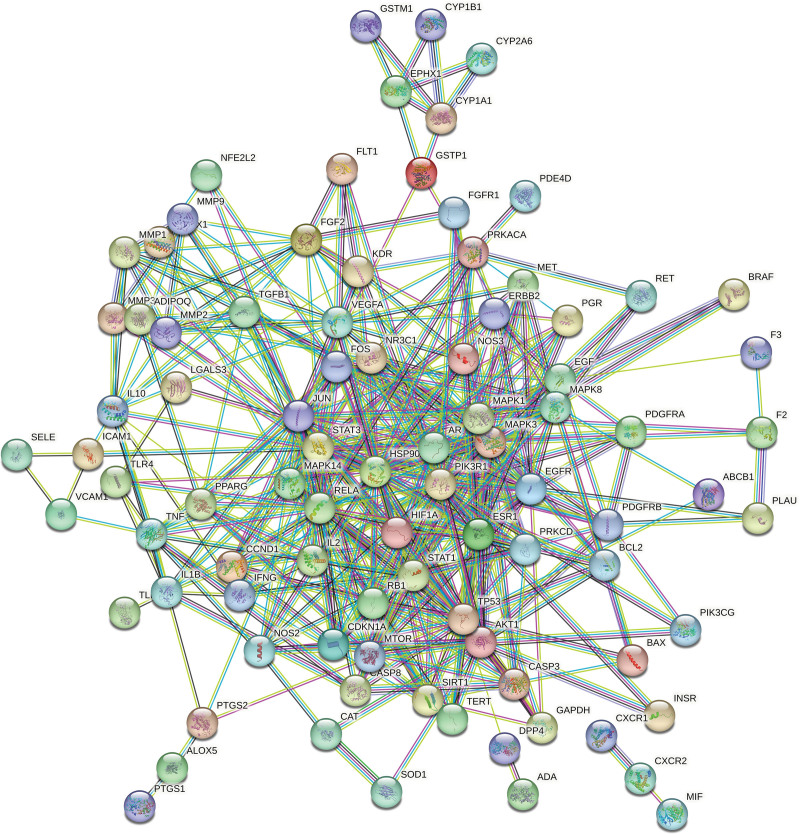
Target protein PPI network diagram. PPI = protein–protein interaction.

**Figure 5.**
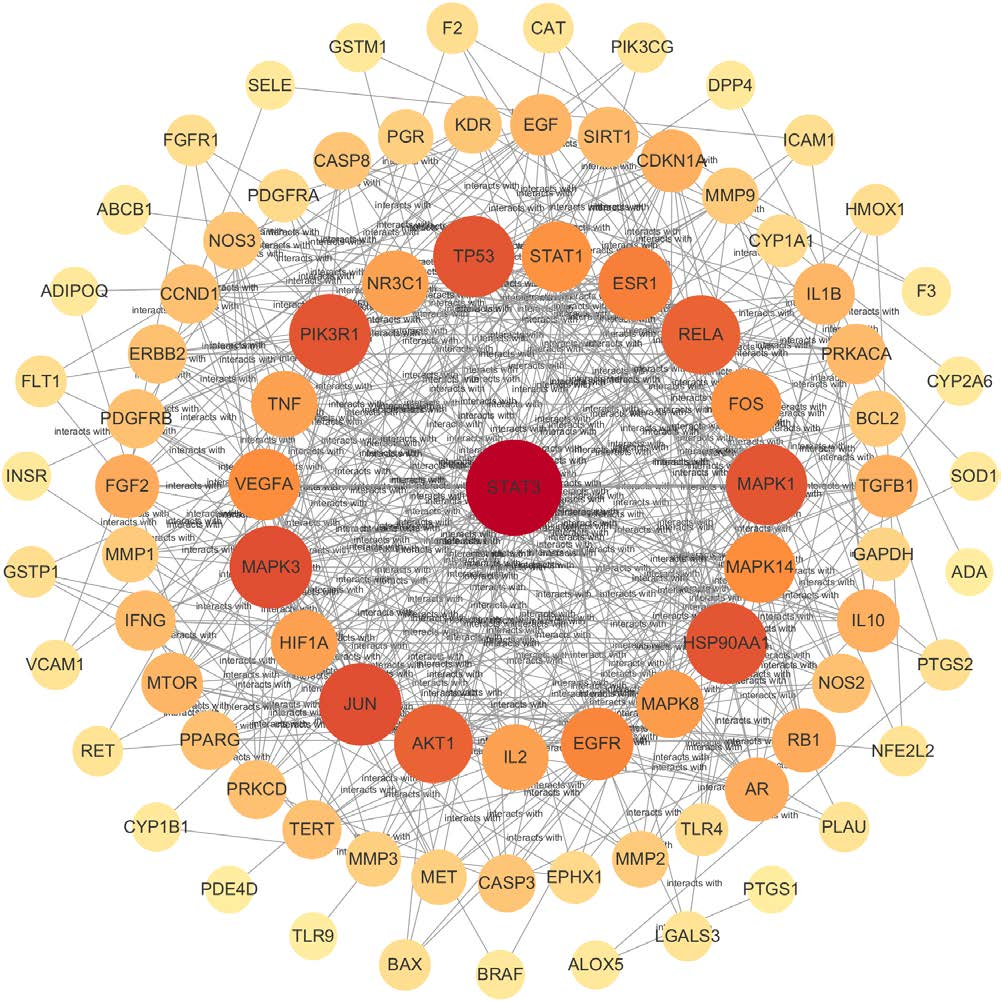
Network map of core targets.

The 178 overlapping target points were imported into the STRING database to construct a PPI network, which comprises 102 nodes and 442 edges, with an average node degree of 8.67 (see Fig. 4). In this network, nodes represent the targets, while edges denote the interactions among them. The data were subsequently imported into Cytoscape version 3.9.1, where nodes with degree, betweenness centrality, and closeness centrality values greater than or equal to the average were identified as key targets (see Fig. 5). Notable key targets for the treatment of pulmonary nodules with WST include signal transducer and activator of transcription 3, Jun proto-oncogene, mitogen-activated protein kinase-3 (MAPK3), mitogen-activated protein kinase-1 (MAPK1), phosphoinositide-3-kinase regulatory subunit 1, heat shock protein 90 alpha family class A member 1, tumor protein p53 (TP53), and AKT serine/threonine kinase 1.

### 3.5. Results of the GO functional enrichment analysis

**Figure 6.**
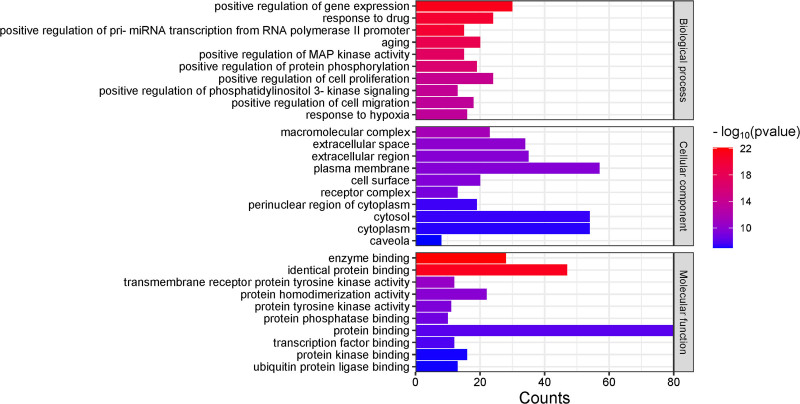
GO enrichment analysis. GO = Gene Ontology.

GO function enrichment analysis, conducted with a *P*-value threshold of <.05, yielded a total of 951 entries. Among these, 728 biological processes were identified, primarily associated with the following functions: positive regulation of gene expression, response to drug exposure, positive regulation of primary microRNA transcription from RNA polymerase II promoter, aging, positive regulation of mitogen-activated protein kinase activity, positive regulation of protein phosphorylation, positive regulation of cell proliferation, positive regulation of phosphatidylinositol 3-kinase signaling, positive regulation of cell migration, and response to hypoxia. Additionally, 112 distinct cellular components were identified, with the majority classified as integral components of macromolecular complexes, extracellular space, extracellular regions, plasma membranes, and cell surfaces. Furthermore, a total of 111 molecular functions were recognized, including enzyme binding, identical protein binding, transmembrane receptor protein tyrosine kinase activity, protein homodimerization activity, and protein tyrosine kinase activity. Arrangement was made according to significance, and the top 10 entries in biological processes, cellular components, and molecular functions were selected as bar plots, as shown in Figure 6.

### 3.6. Results of the KEGG pathway enrichment analysis

**Figure 7.**
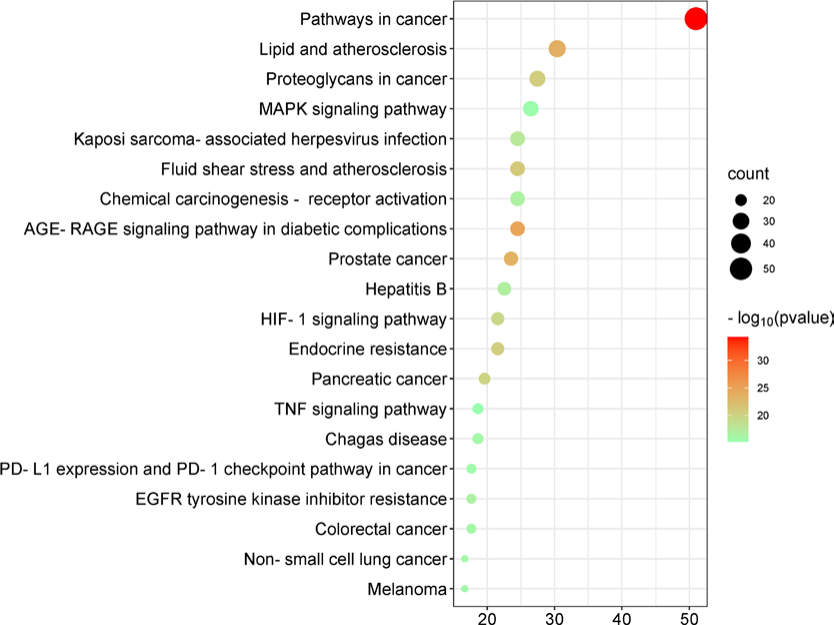
KEGG analysis–bubble plot. KEGG = Kyoto Encyclopedia of Genes and Genomes.

**Figure 8.**
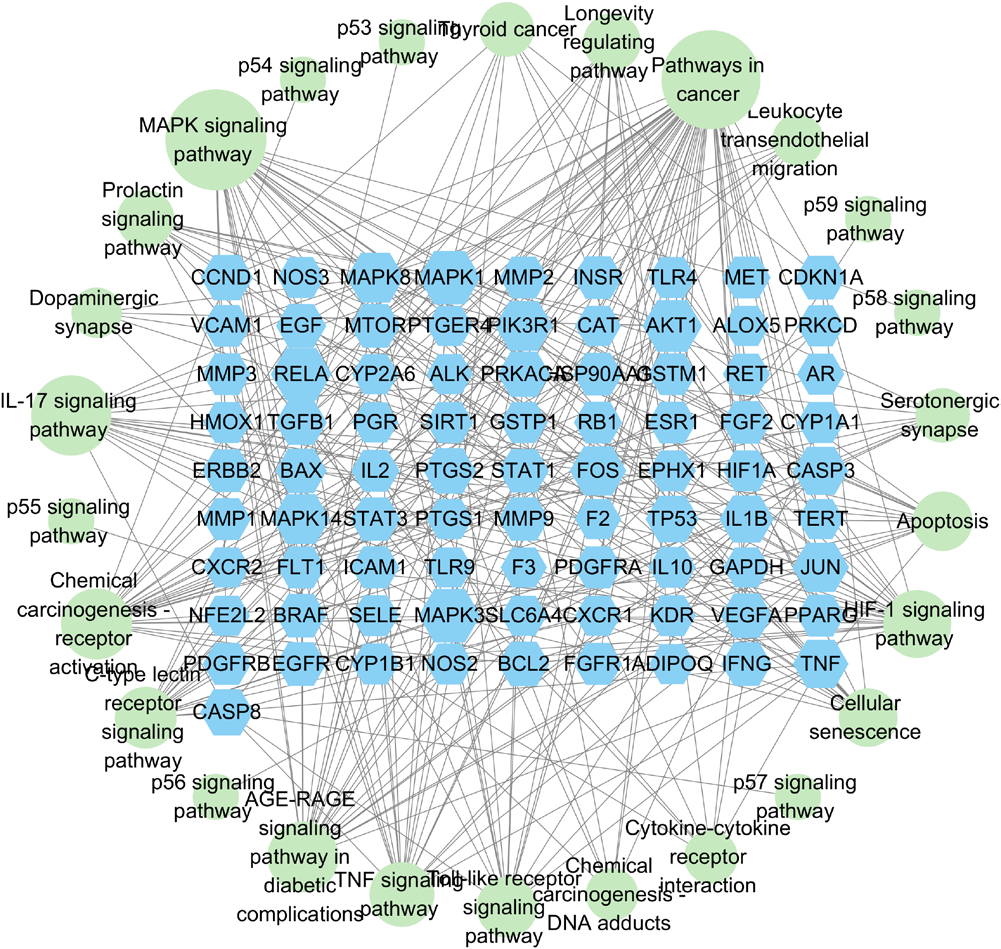
Pathway–target network plot.

KEGG analysis identified enrichment in 163 pathways, which were ranked in descending order based on the Gene Ratio. The top 20 KEGG biological pathways were selected according to their *P* values, with pathway names displayed on the ordinate and Gene Ratios on the abscissa, accompanied by adjusted P values. Bubble plots summarizing these findings are presented in Figure 7. The pathways identified included cancer, the advanced glycation end product (AGE)–receptor for advanced glycation end product (RAGE) pathway, epidermal growth factor receptor (EGFR) signaling, programmed cell death-ligand 1 (PD-L1) pathway, hypoxia-inducible factor-1 (HIF-1) signaling, tumor necrosis factor (TNF) signaling, and mitogen-activated protein kinase (MAPK) pathways. Furthermore, the pathways and their corresponding targets were imported into Cytoscape 3.9.1 to construct a pathway–target network diagram, as illustrated in Figure 8.

### 3.7. Molecular docking

**Figure 9.**
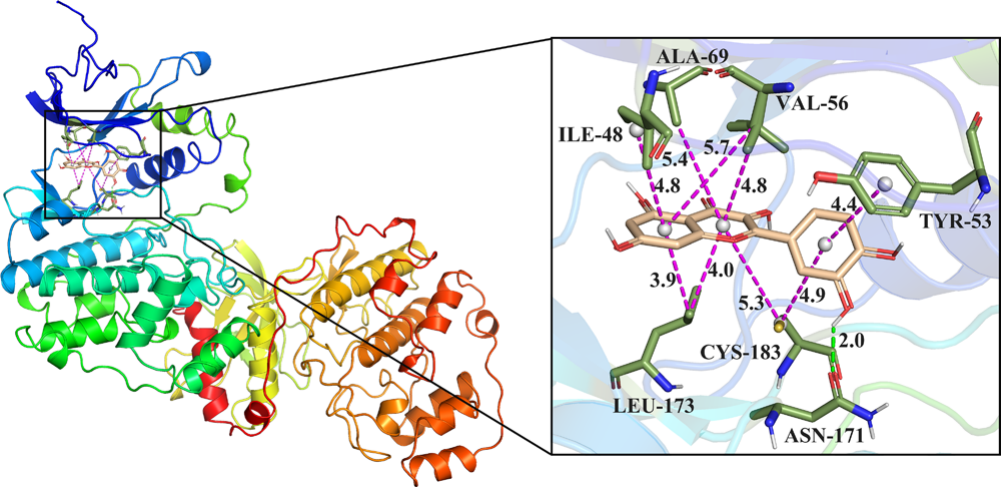
Molecular docking map of quercetin and MAPK3. MAPK3 = mitogen-activated protein kinase-3.

Molecular docking was performed between the key targets identified in the PPI network and the primary active ingredients of WST to evaluate their binding affinities. The binding free energy values were analyzed, with lower values indicating more favorable ligand–protein interactions. A binding energy of less than ‐5.0 kcal/mol suggests good affinity, while a value below ‐7.0 kcal/mol indicates excellent binding activity. The results demonstrated that quercetin exhibited the strongest binding affinity with MAPK3, with a binding energy of ‐9.1 kcal/mol. As shown in Figure 9, the left panel presents an overall view of the docking interaction, while the right panel provides a close-up view. In the diagrams, the light yellow color represents the small molecule, the green dashed lines indicate hydrogen bond interactions, and the purple dashed lines represent hydrophobic interactions.

## 4. Discussion

Pulmonary nodules are the most common radiological manifestation of early lung cancer.^[13]^ The pathological changes between benign and malignant pulmonary nodules exhibit significant differences. Benign lesions are most often granulomas, tuberculosis, hamartomas, intrapulmonary lymph nodes, teratomas, inflammatory pseudotumors, and focal fibrosis.^[2]^ In contrast, malignant lesions predominantly consist of adenocarcinoma, squamous cell carcinoma, small cell lung cancer, and large cell carcinoma, with adenocarcinoma being the most prevalent.^[14]^ The mechanisms underlying the malignant transformation of pulmonary nodules primarily involve abnormal activation of proto-oncogenes, mutations in tumor suppressor genes, neovascularization, dysregulation of immune regulation, abnormalities in preinvasive lesion molecules of the respiratory tract, and mitochondrial dysfunction.^[15,16]^ The pathological changes associated with pulmonary nodules are complex, and the mechanisms underlying their malignant transformation remain incompletely understood. Currently, no specific pharmacological treatments are available, posing significant challenges for early diagnosis and intervention. WST, composed of 10 Chinese herbs, is used for the treatment of pulmonary nodules in China. This study utilized a combination of network pharmacology and molecular docking to explore the potential active ingredients, targets, and mechanisms of action of WST in the treatment of pulmonary nodules.

Through network analysis and degree ranking, the top 5 primary active ingredients of WST were identified as β-sitosterol, stigmasterol, kaempferol, salidroside, and quercetin. β-sitosterol exhibits antitumor and antioxidant biological activities. It inhibits the proliferation of A549 lung cancer cells by blocking the G2/M phase of the cell cycle, while also upregulating Bax and downregulating Bcl-2 expression.^[17]^ Additionally, β-sitosterol induces autophagy in cancer cells through the phosphoinositide-3-kinase/AKT/mammalian target of rapamycin pathway, contributing to its antitumor effects.^[18]^ Stigmasterol, a plant sterol, regulates the expression of apoptotic genes, induces cancer cell apoptosis, and exhibits antitumor, antioxidant, and anti-inflammatory properties.^[19]^ It promotes cancer cell apoptosis by upregulating P53 gene and inhibiting BCL-2 gene, activating the expression of Caspase-8 and Caspase-9 proteins.^[20]^ Kaempferol, a flavonoid compound widely found in nature, serves as a natural sensitizer and anticancer agent for lung cancer treatment. It exerts antitumor effects by inhibiting the expression of the MAPK and phosphoinositide-3-kinase–AKT signaling pathways and inducing autophagy.^[21]^ Salidroside inhibits tumor cell proliferation through multiple pathways including anti-oxidative stress, inhibition of DNA synthesis, induction of apoptosis and autophagy in tumor cells, and inhibition of angiogenesis.^[22]^ Ren et al^[23]^confirmed that salidroside exerts anti-lung cancer effects by upregulating the expression of miR-195 in A549 cells, thereby blocking the phosphorylation of AKT and the mitogen-activated protein kinase kinase/extracellular regulated protein kinases (MEK/ERK). Quercetin exhibits antitumor activity through multiple pathways and mechanisms. It induces autophagy in human lung cancer A549 cells by activating the AMPK–mammalian target of rapamycin pathway.^[24]^ Another animal study^[25]^ demonstrated that quercetin, in combination with suramin, synergistically inhibits the growth of LA795 lung adenocarcinoma xenografts and lung nodule metastasis in T739 mice. These findings suggest that the primary active ingredients of WST may exert therapeutic effects against pulmonary nodules through antitumor, antioxidant, and anti-inflammatory mechanisms.

Further analysis of PPI by Cytoscape 3.9.1 software, the key targets of WST for pulmonary nodules may be signal transducer and activator of transcription 3 (STAT3), Jun proto-oncogene, MAPK3, MAPK1, TP53. STAT3 is activated in lung cancer and plays a crucial role in regulating cell growth, differentiation, senescence, and apoptosis, contributing to tumor progression and immune suppression.^[26]^ A study revealed that in the absence of c-Jun, the protein levels of Jun family member JunD were elevated. Notably, in c-Jun-deficient cells, the phosphorylation of JunD was enhanced, and the expression of a dominant-active JNKK2–JNK1 transgene further promoted lung tumor development. Interestingly, the deletion of JunD completely suppressed rat sarcoma (Ras)-induced lung tumorigenesis.^[27]^ MAPKs are a highly conserved class of serine/threonine protein kinases found within cells. Among them, MAPK3 and MAPK1, also known as Erk1 and Erk2, are key players. Through a 3-tier kinase signaling cascade, activated Erk1 and Erk2 stimulate cell differentiation, proliferation, and migration.^[28,29]^ More than half of the tumors have mutations in TP53 gene. In non-small cell lung cancer, TP53 mutations alter relevant genes involved in cell cycle regulation, DNA replication and damage repair, thus promote the expression of PD-L1, increase the binding of PD-L1 and the receptor programmed cell death protein 1 (PD-1), and ultimately cause immunosuppression.^[30]^ Although pulmonary nodules and tumors are different conditions, they share significant similarities and associations. Many of the targets identified in this study have been previously implicated in lung cancer. Combined with the similarities and associations between lung cancer and lung nodules, it is speculated that the targets of lung cancer may play a similar role on lung nodules. These key targets may thus offer valuable insights for future research on WST in the prevention and treatment of lung cancer.

According to KEGG enrichment analysis, the primary signaling pathways involved include the cancer pathway, MAPK signaling pathway, AGE–RAGE signaling, EGFR, TNF, HIF-1, and the PD-L1/PD-1 checkpoint pathways. Among these, the MAPK signaling pathway is particularly enriched with a large number of genes, and this pathway has a critical role in the growth and proliferation of lung cancer cells, and there are crosstalk associations with other cancer pathways with a high degree of enrichment. Specifically, EGFR overexpression can abnormally activate downstream pathways such as MAPK, which play important roles in biological processes such as cell proliferation and apoptosis, and among the MAPK pathways, Ras/Raf/MEK/ERK is a classically studied signaling cascade associated with tumors, which profoundly influences tumorigenesis and progression.^[31,32]^ Research has shown that nonclassical integrin ITGB2 signaling can activate the EGFR and RAS/MAPK/ERK pathways in small cell lung cancer.^[33]^ Additionally, quercetin, a key component of WST, has been reported to inhibit the metastatic potential of lung cancer by modulating the AKT/MAPK/β-catenin signaling pathway, preventing the intranuclear translocation of β-catenin.^[34]^ The MAPK signaling pathway plays a pivotal role in lung cancer or nodules pathophysiology. Ectopic activation of MAPK3 is known to contribute to various cancers.^[35]^ In this study, molecular docking analysis revealed that quercetin demonstrated the highest binding affinity with MAPK3, with a binding energy of −9.1 kcal/mol. This suggests that quercetin may exert its inhibitory effects on the MAPK signaling pathway by directly interacting with MAPK3, potentially contributing to the suppression of lung nodule progression.

Signaling pathways do not function in isolation. For example, the EGFR signaling pathway is a crucial oncogenic driver and an upstream regulator of the Ras/rapidly accelerated fibrosarcoma 1/MEK/MAPK pathway.^[36]^ TNF can activate the AKT signaling pathway via phosphatidylinositol 3-kinase (PI3-K) and inhibit cytochrome C release in mitochondria-dependent apoptosis.^[37]^ HIF-1 acts as a signaling hub that coordinates the activities of multiple transcription factors and signaling molecules that influence tumorigenesis.^[38]^ Furthermore, PD-L1 is frequently overexpressed in human cancers. PD-L1 binds to the receptor PD-1 on activated T cells and suppresses antitumor immunity by counteracting T cell activation signals.^[39]^ The therapeutic effects of WST on pulmonary nodules involve the modulation of these complex signaling pathways. Notably, quercetin, one of its primary active ingredients, exerts its effects by regulating the MAPK signaling pathway, potentially slowing the progression of lung nodules.

## 5. Conclusion

In conclusion, the primary active components involved in the treatment of pulmonary nodules may include β-sitosterol, stigmasterol, kaempferol, salidroside, and quercetin. The key molecular targets include STAT3, MAPK3, MAPK1, JUN, TP53, phosphoinositide-3-kinase regulatory subunit 1, heat shock protein 90 alpha family class A member 1, and AKT serine/threonine kinase 1. The principal signaling pathways implicated are the cancer pathway, MAPK, AGE–RAGE, EGFR, TNF, PD-L1, and HIF-1 pathways. Among these, key components of WST, such as quercetin, demonstrate considerable potential as therapeutic candidates for pulmonary nodules.

In this study, based on network pharmacology, we explored the mechanism of WST in pulmonary nodules from multiple components, multi-pathway, and verified it through molecular docking technology to provide data support and scientific basis for subsequent studies. However, our study has certain limitations. The structure of WST is complex, and the information of this study is obtained through network database, and the information of database still needs to be further improved. Validity of this study is the lack of experimental support, and its mechanism still needs further experimental verification.

## Author contributions

**Conceptualization:** Guangxi Li.

**Methodology:** Jingqi Yang.

**Writing – original draft:** Jinzhi Zhang.

**Writing – review & editing:** Jinzhi Zhang, Guangxi Li.
